# Indoor measurement and analysis on soil-traction device interaction using a soil bin

**DOI:** 10.1038/s41598-024-59800-2

**Published:** 2024-05-02

**Authors:** Aref Mardani, Behzad Golanbari

**Affiliations:** Department of Mechanical Engineering of Biosystems, Urmia University, Urmia, Iran

**Keywords:** Bevameter, Off-road vehicle, Soil-wheel interaction, Soil stress, Single-wheel tester, Techniques and instrumentation, Mechanical engineering

## Abstract

Investigating the mechanical interaction between vehicles and soil requires measuring various mechanical parameters, which have been enhanced through the advancement of measurement tools. This study delves into the measurement tools and data recording devices utilized in soil-traction device studies conducted at the Off-Road Vehicle Research Center of Urmia University. The tools and methods used for measuring and recording parameters such as forces, velocities, displacements, stresses, and contact areas during traction engagements of laboratory test traction devices (wheels and tracks) are presented. These measurements may have been simultaneously obtained using multiple transducers. The results demonstrate the data acquisition system's successful measurement and recording of these parameters. This research emphasizes the integration of different sensors, developing and implementing a unified data acquisition system for various transducers, and creating innovative setups for measuring and recording parameters such as soil density and wheel-soil contact area, which are of significant importance.

## Introduction

Studying the behavior of soil with tools and off-road vehicles is challenging due to its complexity and unpredictable nature. The interaction between a tire and soil is a topic of great interest among researchers^1^. Despite the complexity of the wheel-soil interaction, various methods have been used to investigate this process^2^. Soil tests are mainly conducted in outdoor and indoor environments^3^. Measurement methods in outdoor environments are easier, but controlling experimental variables can be challenging. On the other hand, indoor measurements provide easier control over experimental variables compared to outdoor measurements^4^. In indoor testing methods, tire-soil tests under controlled conditions are typically performed using wheel test devices employed in a soil bin. These environments offer more control over tire-soil variables. The soil bin is a channel filled with homogeneous soil under uniform conditions. Soil bins can be constructed in various forms. Some types have movable rails within the soil bin, while others are fixed soil channels that may be excavated into the ground or placed as a structure on the ground surface^5^.

A soil bin comprises several components, including the carrier, soil preparation equipment, traction device, drive system, measuring instruments, and control systems^6^. This equipment facilitates the determination of various factors such as stress, strain, traction force, wheel slip, soil and tire deformations, and soil parameters.

In the field of soil-tire interaction, traction testing devices use measurement systems equipped with sensors designed to detect various mechanical parameters. Advancements in technology have replaced the initial methods of measurement in machine-soil interaction studies with transducers based on analog and digital measurements. In 1914, George Kuehne conducted pioneering research on the interaction between machines and soil in a soil bin located in Berlin, Germany^7^. During that time, the measurement equipment used in these studies was predominantly mechanical and relied on basic measurement technologies. In the subsequent years, Mark L. Nichols and John W. Randolph also conducted their research on the mechanics of machine-soil interaction in the soil bin, primarily utilizing early mechanical measurement equipment^8^. Furthermore, government agencies in the USA and other countries have developed various single-wheel testers for this purpose^9^. Noteworthy research facilities dedicated to the study of vehicle-soil interaction have emerged worldwide, including the National Soil Dynamic Laboratory (NSDL) in Auburn, USA, Cranfield University at Silsoe in the UK, the University of California at Davis, USA, the University of Hohenheim in Germany, and IMAG of Wageningen in the Netherlands. These facilities have made significant contributions to the field of soil-machine interaction^9^.

The use of soil bins in research related to soil-machine interaction is of significant importance^10^. A soil bin is a laboratory system designed to enable the investigation, analysis, and characterization of soil behavior in interaction with machinery. This system provides the possibility of simulating different conditions present in soil operations and structures, facilitating the study of the effect of these conditions on soil behavior and machinery performance^11^. By utilizing a soil bin, it is possible to investigate the behavior of soil and its response to machinery loading in a controlled manner, eliminating the need for large-scale experiments. This equipment allow the examination of various factors, including soil type, moisture content, density, loading rate, and machinery performance under controlled conditions. The use of a soil bin can enhance machinery performance under different soil conditions. Additionally, special soil samples can be employed to simulate unique conditions, enabling the study of the performance of special machines such as Mars rovers^12^.

This research represents diverse and independent efforts in Terramechanics and machine-soil contact studies, embodying a synthesis of varied insights and findings amassed over time through parallel research endeavors. Within this article, we meticulously outline the comprehensive sequence of meticulously designed and tested sections, elucidating the measurement techniques employed at each stage and anchoring our methodologies with references to prior research. This compilation stands out within the field of Terramechanics studies for its unique integration of various measuring devices, a feature seldom seen in prior scientific research, making its introduction imperative. By emphasizing the robust infrastructure of this setup, we unlock the potential for comprehensive data acquisition, catering to modern methodologies rooted in artificial intelligence and numerical analysis. As a non-commercial endeavor born from research ideas and innovations, this collection stands as a testament to scientific novelty and achievement, providing a solid foundation for cutting-edge research techniques in this dynamic field. The main objective of this study is to provide a comprehensive examination of different methods and tools used to measure critical parameters in the field of soil-wheel interaction studies, specifically in soil bin environments. By assessing a wide range of measurement tools and techniques, this study enables researchers to evaluate their strengths, limitations, and applications in soil-wheel studies, offering valuable insights into the selection, calibration, and use of measurement instruments to ensure reliable and accurate data collection.

## Material and method

**Figure 1:**
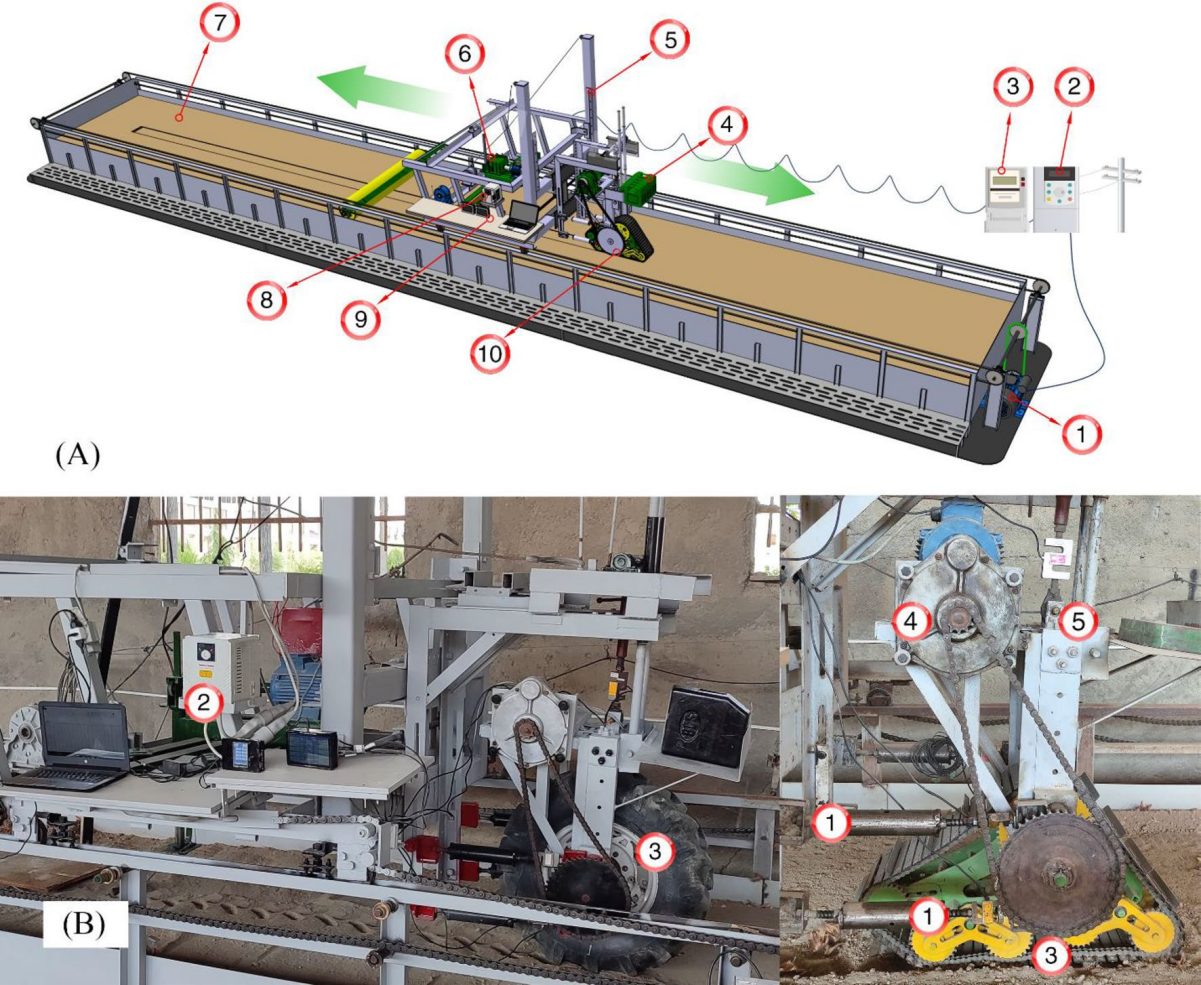
Soil bin with single wheel tester and tire-soil interaction measurement equipment. (**A**) 1. Carrier Electromotor, 2. Carrier Inverter, 3. Three phase power supply, 4. Static load, 5. Adjusting the height of the wheel tester, 6. Bevameter, 7. Soil channel, 8. Single wheel tester wheel/ Bevameter/ Penetrometer inverter, 9. Data logger, 10. Single wheel tester. (**B**) 1. Traction arms with load cell for traction force measurement, 2. Single wheel tester wheel inverter (Electrical speed reduction), 3. Traction devices, 4. Gear box with chain (Mechanical speed reduction), 5. Dynamic load.

A soil bin is a controlled environment used to study the interaction between machines and soil. It typically consists of a soil channel with various dimensions, a carrier for installation and movement along the channel, a power transmission system, and measurement equipment. The soil bin used in this study is a fixed metallic structure placed on the ground surface, with the soil bed completely separated from the ground and designed as a soil channel. Figure 1A illustrates the various components of the soil bin.

The soil bin boasts dimensions ideal for experimentation, featuring a 24 m long channel with a width of 2 m and a soil layer depth of 1 m. This setup provides optimal conditions to conduct a wide array of experiments effectively. The soil within the bin is meticulously chosen to replicate real-world conditions, comprising a silty-loam soil type. This soil texture comprises 35% sand, 22% silt, and 43% clay, ensuring a balanced representation of soil properties. This study does not consider the mechanical structure of the soil bin's different sections but rather concentrates on evaluating the measurement systems in each section. Additionally, it examines the integrated and simultaneous data acquisition system across multiple sections for a comprehensive analysis.

### Tire/track theoretical velocity measuring

The theoretical velocity pertains to the linear speed of traction devices, like pneumatic or track wheels, and may surpass the actual movement speed due to slippage. This speed is attained through a 5.5 kW electric motor serving as the driving force, adjusted via a two-stage reduction process that encompasses both mechanical and electrical reduction (refer to Fig. 1B). Mechanical reduction involves a gearbox, and electrical reduction employs an LS-branded inverter produced by LG in South Korea.

The theoretical speed (*V*_*t*_) of the traction factor is given by Eq. 1 in terms of the feeding frequency.

1 $$V_{t} = \frac{{f_{w} }}{6.82}$$

where, *f*_*w*_ is the feeding frequency of the inverter.

**Figure 2:**
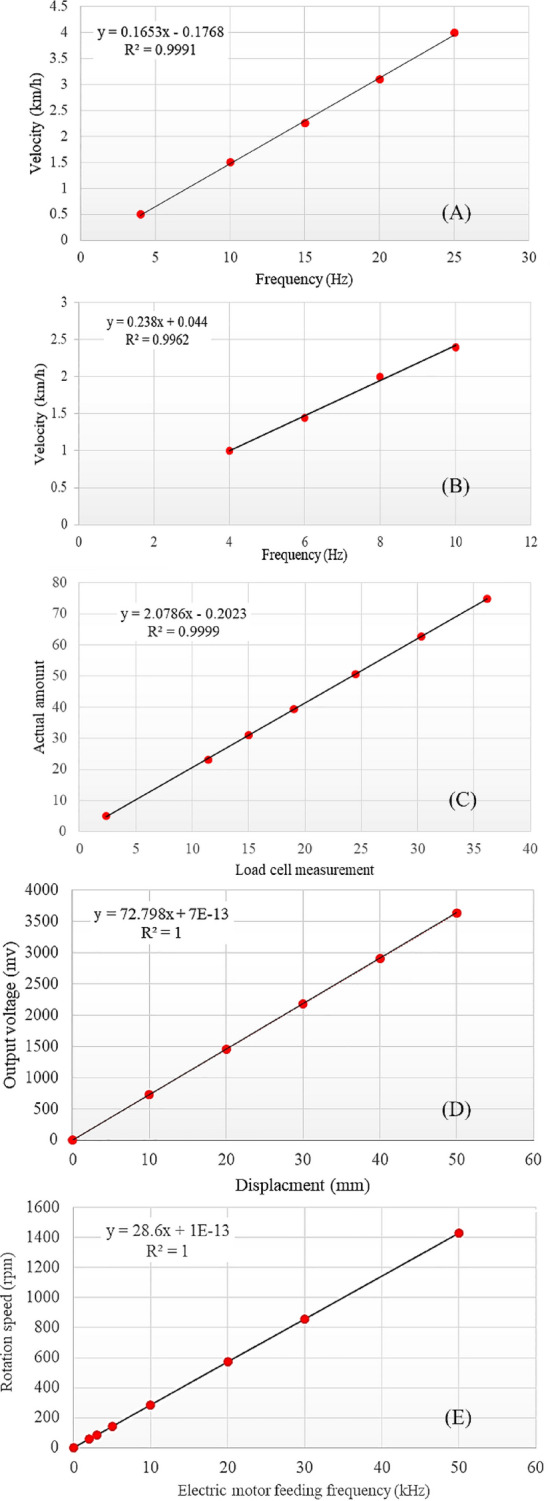
Calibration diagrams of all types of soil bin controllers and sensors with calibration relationships. (**A**) Wheel inverter velocity-frequency diagram, (**B**) Carrier inverter velocity-frequency diagram, (**C**) Graph of actual values with values measured by load cell, (**D**) Calibration graph for differential linear transducer, (**E**) Frequency-rpm diagram by inverter controller.

The calibration graph for the second electric motor inverter is depicted in Fig. 2A. This graph synchronizes the wheel speed with the frequency of the electric motor via the inverter, ensuring that the wheel speed closely aligns with the desired speed.

The measurements and findings presented in this section served as baseline information for a study utilizing an artificial neural network to predict the energy efficiency of driven wheels. This study confirms that precise and reliable measurements are pivotal in any scientific investigation, particularly when analyzing complex phenomena like energy efficiency in driven wheels. The information in this section provides practical insights for assessing wheel performance and optimizing associated factors across various applications.

### Tire/track real velocity measuring

The current velocity of either the pneumatic or tracked wheel aligns with the rate at which the carrier is drawn through the soil channel. To facilitate the operation of the soil channel and supply the necessary power, a 22 kW (30 hp) industrial three-phase electric motor has been employed. This electric motor furnishes the propelling force to mobilize the carrier, subsequently transmitted to the axle at the channel's periphery via the sprocket. The motion is then conveyed to two sprockets positioned on the channel's sides, facilitating dual-sided movement (front and rear) of the wheel carrier along the channel.

Given that speed is a crucial dynamic parameter in vehicle movement, we utilized a 22 kW inverter manufactured by LS, a brand under LG from South Korea, to regulate the rotational speed of the drive motor. This control mechanism directly influences the linear speed of the driven carrier. The inverter boasts a frequency range from 0 to 50 Hz, with a precise step size of 0.01 Hz.

The inverter continuously adjusts and sets the rotational speed of the electric motor by modifying the voltage-frequency combination supplied to it. It offers an unlimited number of speed levels or increments. The utilized inverter is programmable and capable of motion control or motion planning, enabling the creation of movements with user-defined velocity profiles or customized motion patterns. To minimize dependency on the inverter when reducing motor speed, efforts have been made to introduce mechanical modifications in the power transmission path. These modifications aim to achieve a speed close to the desired maximum, regardless of the inverter's influence. Gear ratios have been selected to attain a speed of 6 m/s for the carrier's movement, considering the motor's nominal speed of 1457 rpm, even without the inverter's intervention.

The inverter continually adjusts the rotational speed of the electric motor by modifying the voltage-frequency combination supplied to it. It offers an infinite number of speed levels or increments. The programmable inverter is capable of motion control or motion planning, enabling the creation of movements with user-defined velocity profiles or customized motion patterns. To reduce motor speed with minimal dependency on the inverter, efforts have been made to incorporate mechanical modifications or transformations in the power transmission path. These modifications aim to achieve a speed close to the desired maximum speed, irrespective of the inverter's influence. For this purpose, gear ratios have been chosen so that, even without the inverter's intervention, a speed of 6 m/s can be achieved for the carrier's movement, considering the motor's nominal speed of 1457 rpm. The inverter provides speed adjustments from zero to 6 m/s, finely controllable in deferent increments or levels. In these conditions, the forward speed is linearly related to the inverter's supply frequency and can be determined using Eq. 2 by adjusting the inverter's supply frequency to obtain the actual speed or carrier speed.

2 $$V = \frac{{f_{c} }}{4.34}$$

where, *V* represents the forward speed and *f*_*c*_ represents the frequency supplied to the inverter.

The inverter can implement linear frequency changes to regulate the motor's rotation, ultimately determining the linear speed of the carrier. Figure 2B illustrates the calibration graph depicting the relationship between the frequency transmitted by the inverter and the linear speed of the carrier.

The measurements and findings presented in this section served as the foundation for a study on the impact of speed on the rolling resistance of wheel^13^. This study explores the intricate relationship between the wheel's movement speed and the resistance encountered during rotation. Understanding the influence of velocity on wheel rolling resistance holds significant importance in diverse fields, including transportation, automotive engineering, and tire design. The measurements acquired in this section offer precise and reliable data regarding the wheel's actual speed during operation. By precisely quantifying the actual speed, researchers can establish a robust foundation for analyzing phenomena related to speed.

### Slip measuring

In vehicles, there is always a speed difference between the wheel rotation speed and the forward speed, referred to as slip. Slip denotes the variance between the actual speed and the theoretical speed of a wheel. This slip parameter profoundly influences the traction performance of a vehicle and is directly incorporated into analytical and empirical equations for predicting and calculating traction, as well as traction efficiency. According to traction theory, when there is no slip, the traction generated on the soil would be zero. Slip (*S*) is determined by considering the actual speed and theoretical speed of motion, utilizing Eq. 3.

3 $$S = \frac{{V_{t} - V}}{{V_{t} }}$$

To measure slip, it's essential to determine both the actual and theoretical speeds. In essence, the two electric motors installed on the soil bin operate simultaneously to induce the desired slip on the wheel. The term "actual speed" pertains to the carrier's movement speed, determined by the inverter's feed frequency as calculated using Eq. 4.

4 $$f_{w} = \frac{V}{{0.14\left( {1 - S} \right)}}$$

**Figure 3:**
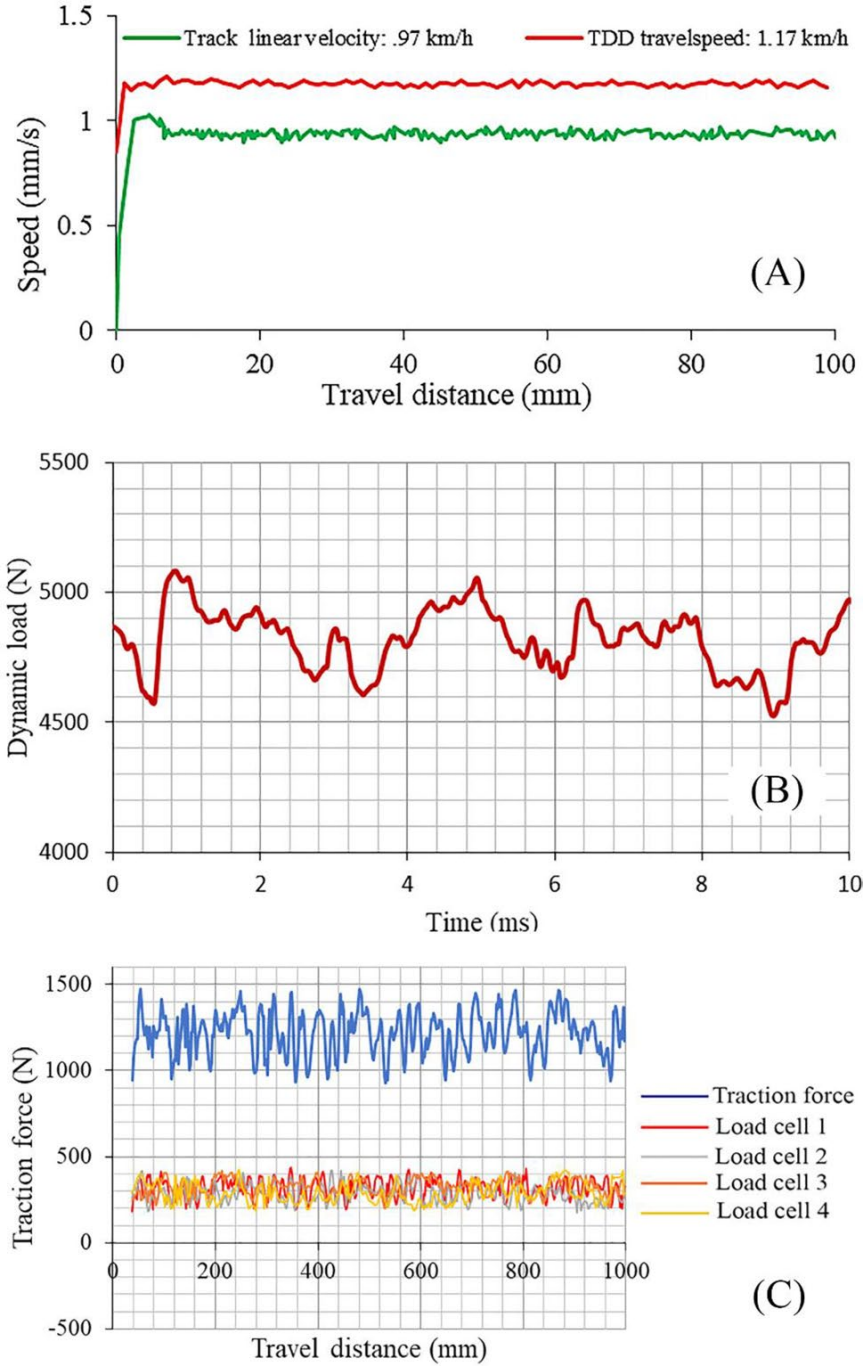
(**A**) The linear speed of the output of the wheel carrier electric motor and the wheel electric motor, (**B**) Dynamic load measuring using S-shape vertical load cell, (**C**) Total traction force and the measured traction force by load cells on the traction arms.

This configuration enables the application of various slip percentages to the wheel. For instance, in Fig. 3A, the linear output speeds of the carrier's electric motor (actual speed) and the startup speeds of the tracked wheel tester's electric motor (theoretical speed) are depicted in a real measurement corresponding to the applied frequency values of the inverters. The illustration highlights that the tracked wheel tester is configured to produce a 17% slip.

The accurate measurement of base slip formed the foundation of Taghavifar and Mardani's study (2015a)^14^, which aimed to explore the impact of slip and speed on the net drive wheel traction. This study primarily focused on measuring slip and ensuring the precise collection of data for a comprehensive analysis. Researchers employed techniques and measurement tools introduced in this section, specifically designed for obtaining base slip data, to ensure the reliability of their findings.

### Dynamic load

The measurement of the vertical load applied to a traction device is crucial for understanding a vehicle's mechanical state and its interaction with the soil. Various factors influence this measurement, including static load and dynamic conditions during vehicle movement. To accurately assess these forces, ensuring the measurement's quality is paramount. Depending on the specific study on soil-wheel (or track) interaction, the system undergoes different loadings, encompassing both static and dynamic scenarios.

To apply a range of dynamic load values on the wheel as experimental treatments, we employ a power screw mechanism (Fig. 3B). The power screw's length is adjusted to create and apply the desired force on the wheel. At each moment, we measure the force using an S-shaped load cell with a carefully chosen nominal capacity suitable for the experiments. The measured force is then transmitted to the data acquisition system. It's essential to note that the employed measurement system ensures a high level of accuracy, with a precision of up to 1 N, allowing us to record and observe the applied force with great reliability.

In this research, the method outlined in this section was employed to explore the impact of dynamic loads on rolling resistance^15^.

### Traction force

From the perspective of Terramechanics studies, the significance of the traction device cannot be overlooked. When we neglect the acceleration at the beginning and end of the motion, the net traction generated by the traction device becomes the cumulative outcome of the forces acting on the horizontal arms attached to the frame.

In the experimental setup, the traction device frame is upheld by four horizontal arms, with each arm linked to an individual load cell, as illustrated in Fig. 1B. The force in each arm is continuously monitored and recorded by a Data Logger, ensuring precision and consistency in data collection.

To underscore the precision of the measurements, the four forces acquired from these load cells undergo meticulous processing and integration to determine the net traction. For a tangible illustration, Fig. 3C displays the total traction value derived by summing the data from these four horizontal load cells. The method presented for measuring traction in this section has found widespread application in numerous previous research studies^14,16,17^. Over the years, researchers have employed this method to investigate and analyze traction performance across various applications. The consistent utilization of this method in different studies has yielded a wealth of supporting evidence, affirming the accuracy, reliability, and repeatability of the measurements. Results from these prior studies consistently demonstrate the method's capability to accurately capture and quantify traction performance. Measurements obtained through this method have proven highly accurate, providing valuable insights into the relationship between different variables and the resulting traction characteristics.

### Soil stress measuring

The measurement of soil stress is crucial for agricultural soil management and understanding the mechanics of soil-machine interaction. Soil stress directly influences the traction force applied by traction devices, making it a valuable parameter for predicting traction and effectively managing machinery. Worldwide, numerous research studies have aimed to record and measure soil stress at different depths, employing various systems. To ensure the reliable measurement of stress at specific depths, our approach utilizes probes attached to load cells in direct contact with the soil. In several studies^18–20^, this method has been consistently employed to measure stress at various soil depths.

**Figure 4:**
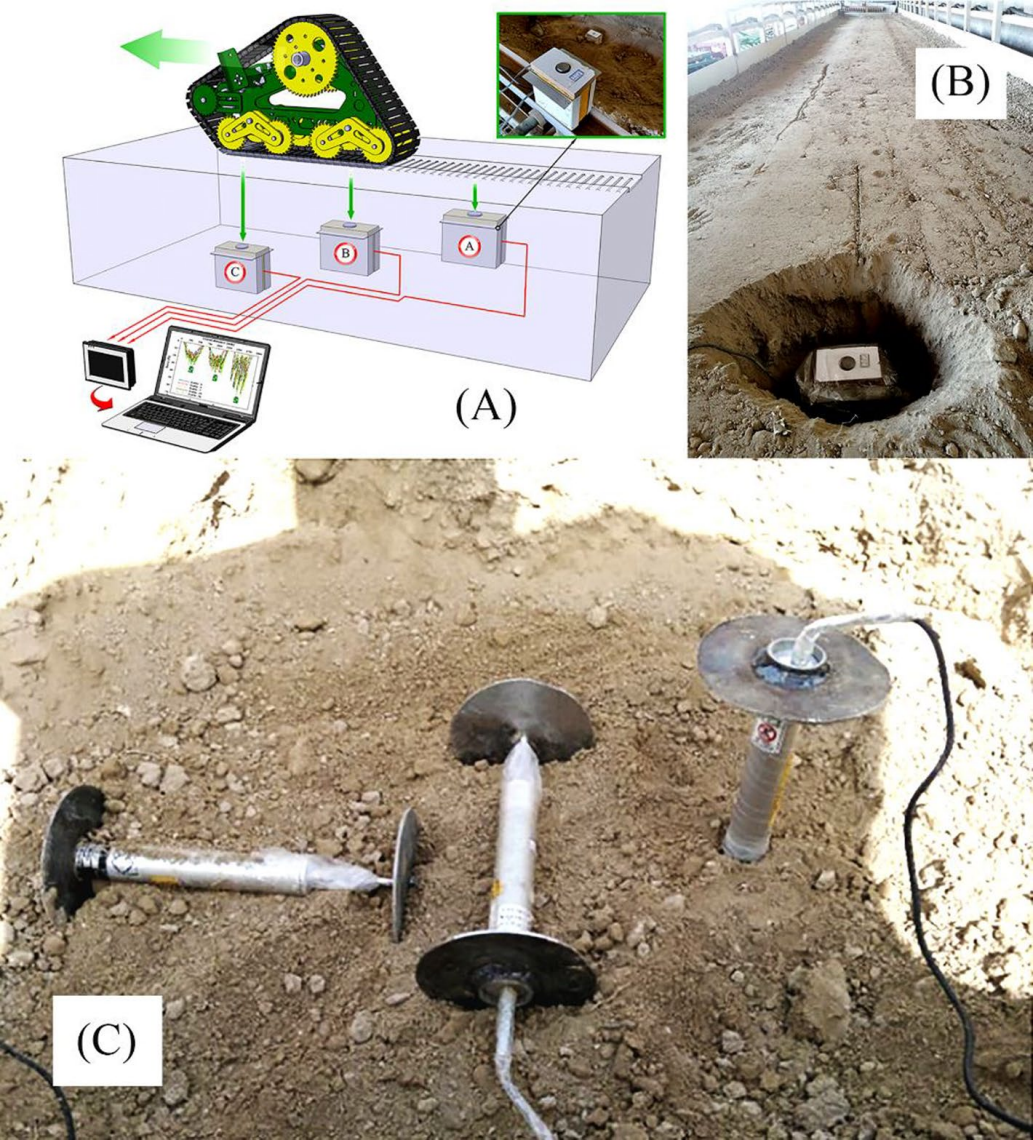
(**A**) Placement of the load cells in the soil, (**B**) Burial of a sample load cell, (**C**) The arrangement of displacement transducers in three coordinate directions inside soil profile.

The load cells utilized have a capacity ranging from 100 to 1000 kg and are Bongshin models manufactured in South Korea. These cells enable the recording of dynamic soil stress behavior at controlled time intervals, both preceding and following the passage of the traction wheel. This meticulous process permits a thorough examination of stress variations over time, aiding in the generation of 3D stress distribution maps around the tire. The installation and positioning of load cells at various depths in the soil are illustrated in Fig. 4A, while the burial of a sample load cell is demonstrated in Fig. 4B.

Calibration of the load cells is an important step that significantly impacts the accuracy of measurements and the reliability of output data. This process ensures that the measurements provided by the load cell are accurate and reliable and reduces the possibility of errors in data collection and analysis. The calibration of the load cells was done before placing them in the soil and in the laboratory. For this purpose, seven different known weights were used, which were previously available. Loading was carried out up to 70% of the nominal capacity of the load cells, and the results were graphically plotted. After conducting field measurements, the obtained results were compared with the calibration data, and the actual values were determined. The calibration graph and the calibration relationship are shown in Fig. 2C.

The stress measurement method presented in this section is a widely recognized and extensively employed approach for measuring stress in soil depth^18,21^. It has been utilized by researchers on numerous occasions, attesting to its popularity and effectiveness in the field of Terramechanics.

The repeated use of this method in previous research studies has yielded strong evidence supporting its accuracy, reliability, and applicability. Researchers have conducted validation studies, cross-referencing the results obtained through this method with data derived from laboratory testing, field instrumentation, or numerical simulations. These comparative analyses consistently demonstrate strong agreement and correlation, confirming the reliability and accuracy of the calculated stress measurements.

### Tire contact area

**Figure 5:**
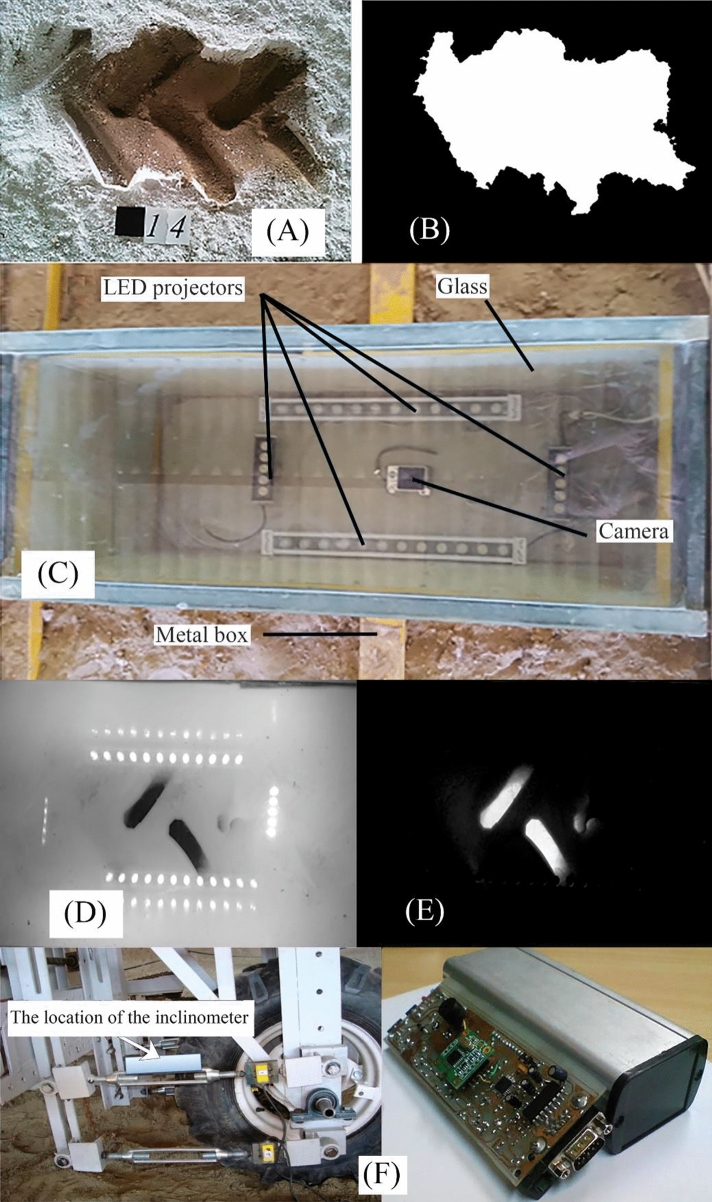
(**A**) A sample of contact area image, (**B**) Thresholding proses to obtain the final analyzed image, (**C**) The glass box and imaging parts of the light project, (**D**) The picture taken from the inside of the box from the tire footprint (**E**) Image analyzed using image processing method to determine contact surface, (**F**) Inclinometer and its location on the traction arm to measure rut depth.

The significance of contact area in influencing vehicle traction, soil compaction, and other soil-tire interaction parameters has been well-established. Measurements of contact area are conducted under both static and dynamic conditions, utilizing various measurement methods. In static contact area measurement between the wheel and soil, the surrounding contact area is entirely covered with gypsum powder, and the wheel is vertically lifted from the soil. The elevated surface is then photographed from a specific distance. As depicted in Fig. 5A, a square marker with a side length of 40 mm is positioned adjacent to each image. Subsequently, image-processing techniques are employed to determine the contact area^22^.

For calibration purposes, we utilized images of a rectangular calibration target with precise dimensions (refer to Fig. 5). The use of this target helps prevent systematic errors in image processing. One common error in image processing research is associated with the type, position, and angles of image capture. To ensure accurate measurements, any camera angles or distortions in the images may result in significant errors. To mitigate such measurement errors, imaging was conducted from a precisely specified and accurate distance while maintaining a perpendicular orientation to the surfaces, as illustrated in Fig. 5A. After capturing the images, they were loaded into MATLAB software, and the calibration element was identified. The software then calculated its pixel units (Fig. 5B). These initial measurements, specifically related to the calibration element, ensure accurate surface contact measurements. To determine the contact surface area, the pixel scale was converted to surface units through a calculation. Lighting conditions may introduce errors at this stage, leading to pixel misalignment across various images and, consequently, impacting surface area calculations. To mitigate this issue, we employed consistent artificial lighting during imaging. Before computing surface contact areas, measurements were meticulously taken to ensure data reliability. Images of various geometries (rectangles, triangles, circles) with known and measured areas were prepared, loaded into the software, and then compared with the calculated areas. The calculated areas by the software matched the measured areas, indicating the reliability of both the software and the image processing algorithm in performing measurements.

The measurement of the contact area between a tire and rigid surfaces has been performed in the Off-Road Vehicles Laboratory at Urmia University. For this purpose, a metal box is used to measure the contact area of the wheel on a solid surface. The box is fixed inside a soil channel (soil bin channel) along the wheel path. The upper surface of the box is made of a 20 mm thick glass plate with dimensions of 130 by 50 cm, and the height of the box is 40 cm. A digital camera is placed at the bottom of the box to capture videos and photos when the wheel passes over the glass. LED lamps are positioned on the four sides of the camera to provide sufficient illumination during the imaging process and ensure image quality uniformity in the software processing stage. These lamps emit light towards the contact area between the wheel and the glass surface during data collection (Fig. 5C).

The glass surface is covered with a white liquid layer with a thickness of 3 mm. As the wheel traverses this liquid layer and comes into contact with the glass, the contact area between the wheel and the glass surface becomes visible. The resulting image combines the white background with dark regions, depicting the contact area. Image processing techniques are then employed to analyze the captured images and derive the dynamic contact area. Derafshpour et al. (2019)^20^ conducted research in this field using the mentioned method. Figure 2C illustrates images before and after processing. In Fig. 5D, the coordination between images before and after processing is clearly visible. The image processing method and its calibration resemble the approach used for analyzing tire deformation on deformable surfaces (Fig. 5E).

The accuracy of the measurements has been validated by assessing test surfaces with measurable areas through geometric methods and comparing the results with image processing methods. The same method is employed to calibrate the measurements for this purpose. Before conducting the main experiments, three calibration plates with known dimensions were utilized. These tests were carried out with multiple repetitions to ensure high accuracy, repeatability, and reliability of the output data using this method. The results obtained from both measurement methods exhibited good agreement with each other.

These contact area measurement methods have been used in Derafshpour et al. (2019) and Tomaraee et al. (2015)^20,23^ studies.

### Rut depth measuring device

The vertical displacement or deformation of the soil is a crucial parameter during soil-machine interaction. This parameter is associated with the resistance to the movement of the traction device and the resulting mechanical changes in the soil, both of which are highly significant. To quantify the vertical displacement in the studied soil bin setup, an inclinometer module (refer to Fig. 5F) was utilized. This device is connected to one of the parallel arms attached to the traction frame and measures the vertical displacement of the traction force on the soil in terms of the change in the angle of the arm relative to a reference axis. The measurements are then sent to the data acquisition system online^24^.

To ensure the accuracy of the inclinometer module measurements and enhance their reliability, an additional method was employed to measure rut depth. A digital caliper (Mitutoyo-model 500-151-30, manufactured in Japan) with a precision of 0.01 mm was utilized to measure sinkage depth following each instance of traffic. First, a T-shaped beam was constructed, and a hole was created in its center. This hole allows the depth measurement blade of the caliper to pass through, and it enables the caliper to be positioned perpendicular to the surface. The T-shaped beam was then affixed to the soil bin chassis, ensuring that the measurement height remains constant. The measurement with this method is done in two steps. In the first step, the height between the beam and the intact surface is measured, and in the second step, the height between the beam and the u rut depth is measured. After taking these measurements, the rut depth is determined by calculating the difference between the two measured values. The beam was designed in a T-shape to minimize deflection caused by its own weight. The measurements were repeated at least three times during each pass to mitigate random errors. In the research conducted by Mardani in (2014)^24^, this method was employed to measure rut depth, confirming its accuracy and reliability.

### Soil deformation measuring

A differential linear transducer, specifically an LVDT (model DTH-A-50) with a maximum displacement of 50 mm, was employed to measure soil displacement. To achieve this, the LVDTs were positioned in the three primary directions: x, y, and z. For precise soil displacement measurement, two plates were affixed at the transducers' endpoints to convey soil pressure or tension to the transducers (refer to Fig. 4C).

The differential linear transducer sends a specific voltage to the sensor output for each displacement value. It can be calibrated by measuring displacement-voltage values (Fig. 2D). A digital caliper (Mitutoyo-model 500-151-30, made in Japan) with an accuracy of 0.01 mm measured displacements in the differential linear transducer. This step aimed to confirm the sensor's displacement accuracy. Subsequently, the voltage for each displacement was recorded, and the corresponding points on the sensor's calibration diagram were plotted. The regression of the resulting graph equaled 1, confirming high measurement accuracy and reliability.

The study conducted by Shahgholi and Abuali (2015)^25^ was centered on investigating soil compaction caused by wheel load using linear differential transducers. In this research, the measurement method outlined in this section was employed to quantify and precisely analyze the effects of the wheel on soil compaction. By embedding these transducers in the soil profile, the researchers could ascertain both vertical and horizontal deformations, along with soil compaction induced by the wheel load.

### Soil properties measuring

In wheel-soil interaction models, it is necessary to calculate the soil stiffness parameters initially. The soil stiffness is a characteristic that describes the soil condition and is expressed through multiple parameters in each model. For example, in models based on the Bekker equation (Eq. 5), the soil stiffness is characterized by three parameters: *K*_*c*_, *K*_*φ*_, and *n*.

To obtain the soil parameters before initiating wheel movement in any given path, at least two plates of different widths are required, and for each plate, three various forces are applied with same sinkage.

5 $$P = \left( {\frac{{K_{c} }}{b} + K_{\varphi } } \right).Z^{n}$$

**Figure 6:**
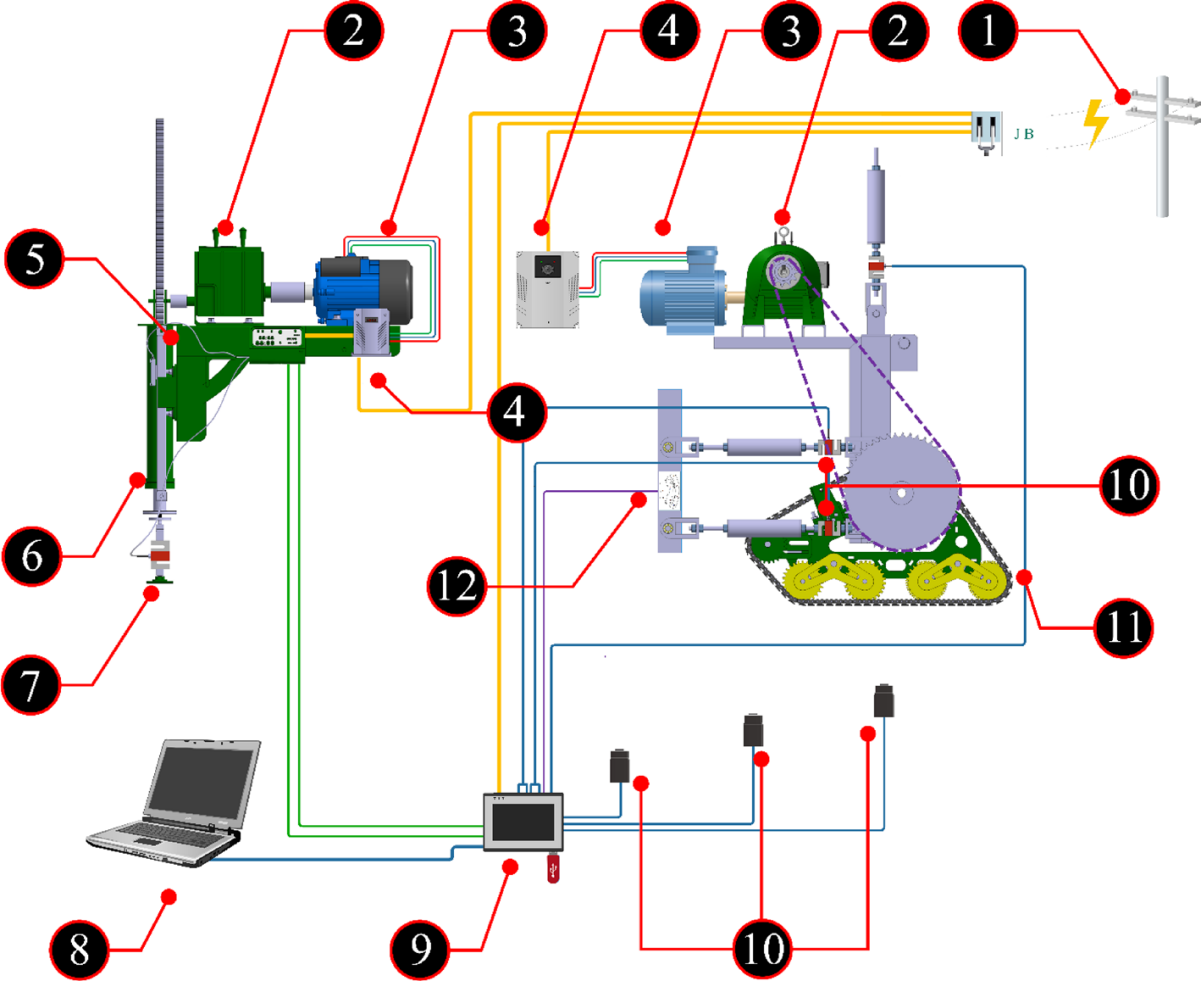
Schematic of data acquisition system. 1. Electric power supply, 2. Gearbox, 3. Electromotor, 4. Inverter, 5. Bevameter (soil properties measuring system), 6. Magnetic linear encoder, 7. Bekker`s Plate, 8. Computer, 9. Data logger, 10. Load cell, 11. Traction device, 12. Inclinometer.

By plotting relevant graphs, one can determine the parameters for each tested soil section. These experiments employ a tool known as a "Bevameter" (refer to Fig. 6). The device has plates installed, and the necessary vertical force for plate penetration into the soil is applied by rotating an electric motor. This rotation is then transmitted to a gearbox and a rack, ultimately reaching the pressure plate through a jack shaft. Simultaneously, the load cell and linear encoder (Digital Readout Scale) record the force–displacement values in the data logger.

For example, if the desired linear speed for plate penetration is equal to 15 mm/s and a gearbox conversion factor of 19 is used. First, the rotation speed of the electric motor corresponding to the linear speed should be calculated, which is calculated from Eq. 6.

6 $$V = \frac{75 n}{{60{\text{ i}}}}$$

where *V* is the actual linear speed and, *i* is the reduction factor of the gearbox and, *n* is the rotational speed of the electric motor in rpm. By substituting the assumed values into the equation, the rotation speed of the electric motor equals 228 rpm. Once the rotational speed is determined, refer to Fig. 2E for the corresponding frequency adjustments.

To conduct Bekker tests and obtain essential soil parameters, we employed a specialized tool called the Bevameter. The Bevameter plays a crucial role in the testing process by enabling accurate and reliable measurements. Figure 6 provides a detailed representation of the internal components and structure of the Bevameter, presenting an exploded view of the device.

The Bevameter operates by measuring two parameters. The first parameter is the vertical force applied to the plates, measured using S-type load cells. The second parameter is the linear movement of the plates, measured with a linear magnetic encoder. A caliper (Mitutoyo-model 500-151-30, made in Japan) with an accuracy of 0.01 was employed to assess the measurement accuracy of the magnetic linear encoder and measure its displacement. To verify the accuracy of the magnetic linear encoder's measurements, the slider was positioned at different displacement points. The distance from the slider to the beginning of the main rail was then measured using a caliper. Simultaneously, data concerning the slider's displacement (as measured by the encoder) is shown on the data logger screen. The accuracy of the encoder's measurements is established by comparing the caliper measurements with the values displayed on the screen.

### Data acquisition system

To conduct statistical analysis and assess the impact of variables on experimental results, it is essential to store the measured data persistently for each experiment. The digital data loggers employed possess the capability to connect with up to 8 parallel load cells, offering cumulative output. Each display features an RS232 port for independent transfer to a computer. To ensure the simultaneous transfer and recording of output data from all load cells, a dedicated interface is employed for data transmission to the computer. The system features eight input channels, each capable of recording data at a frequency of 60 Hz, as illustrated in Fig. 6.

### Safety

To ensure the safety conditions of the setup, conservation interventions have been considered. These interventions are generally implemented in two parts: mechanical and electrical. An earth connection has been employed to prevent the risk of electrical shock. Emergency power cutoff switches are installed at various points in the soil bin. Microswitches, connected to both the control system and the electrical panel of the soil bin, ensure the carrier stops at the end of the channel. This guarantees that, in case of any delay by the operator in stopping the carrier, the microswitches will activate promptly.

### Permission for land study

The authors declare that all land experiments and studies were carried out according to authorized rules.

## Conclusion

In this study, a soil-machine interaction test system is introduced, which comprises hardware and data acquisition and processing system. Such sets are typically not commercially available and are developed and expanded in a research context. The setup introduced at Urmia University has the capability of coupling soil and wheel instruments, as well as traction devices and tire segments. In addition to introducing some applications of the set, various transducers along with measurement considerations such as calibration and data logging are presented in this report. Wheel traction parameters such as traction, slip, forward velocity, and rolling resistance, along with some soil parameters such as deformation, density, and mechanical properties of the soil, are among the quantities that are predicted to be measured and recorded in this set.

## Data Availability

The data supporting the findings of this research article are available upon request. The authors are committed to promoting transparency and reproducibility in scientific research. To request access to the data, interested parties may contact the corresponding author directly. The authors will make every effort to provide the data in a timely manner, considering any legal, ethical, or privacy restrictions that may apply. By sharing the data, the authors aim to facilitate further analysis, validation, and collaboration in the scientific community.
